# Characterization of the complete plastid genome of of *Veronica eriogyne* H. Winkl., a Tibetan medicinal herb

**DOI:** 10.1080/23802359.2021.1998802

**Published:** 2021-11-12

**Authors:** Qupei Danzeng, Renqian Suonan, Qien Li, Ciren Nima

**Affiliations:** a Beijing University of Chinese Medicine, Beijing, P. R. China; b Tibetan Medical Hospital of Qinghai Province, Xining, P. R. China; c Tibetan Medicine Research Center, Tibetan Medical College, Qinghai University, Xining, P. R. China

**Keywords:** Evolutionary analysis, Oleaceae, plastid genome, *Veronica eriogyne* H. Winkl

## Abstract

*Veronica eriogyne* H. Winkl.(Plantaginaceae) is a perennial herb with high medicinal value. To better understand the molecular genetics and evolutionary of *V. eriogyne*, its complete plastid genome was sequenced and annotated. The assembled chloroplast genome is a circular 151,083 bp sequence, consisting of a 82,302 bp large single copy region (LSC) and a 17,449 bp small single copy region (SSC), which were flanked by a pair of 25,666 bp inverted repeats (IRs). The GC content of the chloroplast genome is 38.03%. Moreover, a total of 134 functional genes were annotated, including 88 protein-coding, 38 tRNA, and 8 rRNA genes. Phylogenetic analysis showed that *V. eriogyne* has close relationship with *V. persica* Poi. The current study provides important information for further genetic studies on Plantaginacea.

*Veronica eriogyne* H. Winkl. is a perennial herb of the family Plantaginaceae (Hong et al. 1998; Müller & Albach 2010). As a Tibetan medicinal herb, the whole plants of *V. eriogyne* H. Winkl. have the obvious effects of absorb clots, heat-cleaning and detoxifying, analgesia, hemostasis, sterilization, healing sore, antihypertensive, etc. (Cui et al. 2015; Chen and Ge-Sang 2017). Plastid genomes are highly conserved in sequence and structure due to their haploid, non-recombinant, and uniparentally inherited nature (Wicke et al. 2011). Nonetheless, variations within plastid genomes had been revealed in many angiosperm lineages, and can provide abundant evolutionary information (Cosner et al. 2004; Li et al. 2021). Here, the complete plastid genome of *V. eriogyne* H. Winkl. was obtained base on Illumina sequencing data and a evolutionary analysis of *V. eriogyne* H. Winkl. and its allies was carried out.

The samples of *V. eriogyne* were collected from Qunjia Township, Huangzhong County, Qinghai Province, China (36.27°N, 101.68°E). The DNA was extracted from fresh leaves (about 0.2 g) with a modified CTAB method. The voucher specimen and DNA (Specimen accession number: LQE-2019-069; https://zyxy.qhu.edu.cn/jgsz/jxkysw/zyyyjzx/index.htm, Qien Li, qienli@qhu.edu.cn) were kept at the Specimen Room of the Tibetan Medicine Research Center of Qinghai University. Whole-genome sequencing were conducted by Novogene Co., Ltd. (Tianjin, China) with the Illumina NovaSeq 6000 Sequencing System (Illumina, San Diego, CA, USA). Approximately 4 GB of clean data were yielded (GenBank accession no. MH394402). SPAdes v3.10.1 (Bankevich et al. 2012) was used to assemble the plastid genome. The plastid genes were annotated with CPGAVAS2 (Shi et al. 2019).

In order to reveal the phylogenetic position of *V. eriogyne* H. Winkl. with its close relatives of Plantaginaceae, a total of 18 complete plastid genome sequences were involved in a evolutionary analysis. The complete sequences were aligned by MAFFT version 7.473 (Katoh and Standley 2013), and evolutionary analysis were conducted in MEGA7 (Kumar et al. 2016). The bootstrap percentages of trees in which the associated taxa clustered together based on 1000 replicates are shown at the branch nodes.

The complete plastid genome of *V. eriogyne* H. Winkl. was 151,083 bp in length and its GC content was 38.03%. This plastid genome has a typical quadripartite structure, containing a pair of IR regions of 25,666 bp, an LSC region of 82,302 bp, and an SSC region of 17,449 bp. The two IRs were separated by the LSC and the SSC. A total of 134 functional genes were annotated, including 38 tRNA, 8 rRNA and 88 protein-coding (mRNA) genes. The tRNA, rRNA, and protein-coding genes account for 28.36, 5.97, and 65.67% of all annotated genes, respectively. According the result of phylogenetic analysis (Figure 1), *V. eriogyne* H. Winkl. has close relationship with *V. persica* Poir. The current study provides important information for further genetic studies on Plantaginacea.

**Figure 1.**
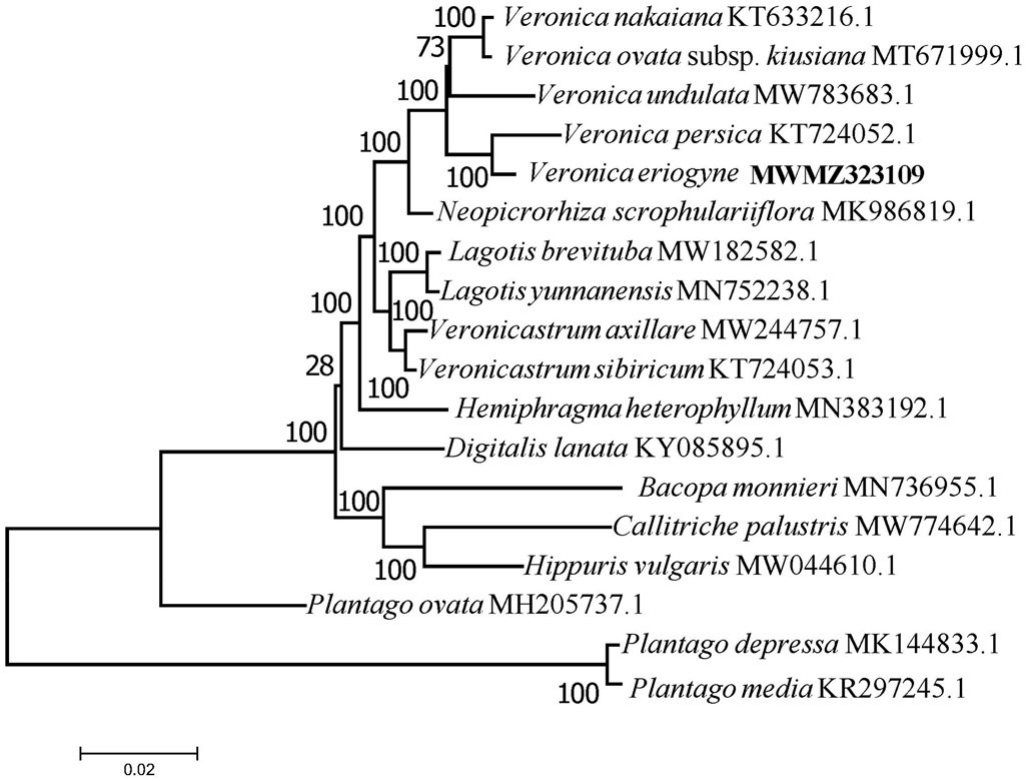
ML tree based on the complete plastid genome sequences. Numbers above branches are bootstrap percentages (based on 1000 replicates).

## Data Availability

The genome sequence data obtained in this study is openly available in GenBank of NCBI at https://www.ncbi.nlm.nih.gov/ under the accession number MZ323109. The associated BioProject, SRA, and Bio-Sample numbers are PRJNA733027, SRR14664914, and SAMN19357950, respectively.
